# Application of Spatiotemporal Hybrid Model of Deformation in Safety Monitoring of High Arch Dams: A Case Study

**DOI:** 10.3390/ijerph17010319

**Published:** 2020-01-02

**Authors:** Chongshi Gu, Xiao Fu, Chenfei Shao, Zhongwen Shi, Huaizhi Su

**Affiliations:** 1 State Key Laboratory of Hydrology-Water Resources and Hydraulic Engineering, Hohai University, Nanjing 210098, China; csgu@hhu.edu.cn (C.G.); chenfeishao.hhu@gmail.com (C.S.); shizhongwen@hhu.edu.cn (Z.S.); su_huaizhi@hhu.edu.cn (H.S.); 2 College of Water Conservancy and Hydropower Engineering, Hohai University, Nanjing 210098, China; 3 National Engineering Research Center of Water Resources Efficient Utilization and Engineering Safety, Hohai University, Nanjing 210098, China

**Keywords:** deformation analysis, data representation, spatiotemporal hybrid model, hydraulic component, finite element method

## Abstract

As an important feature, deformation analysis is of great significance to ensure the safety and stability of arch dam operation. In this paper, Jinping-I arch dam with a height of 305 m, which is the highest dam in the world, is taken as the research object. The deformation data representation method is analyzed, and the processing method of deformation spatiotemporal data is discussed. A deformation hybrid model is established, in which the hydraulic component is calculated by the finite element method, and other components are still calculated by the statistical model method. Since the relationship among the measuring points is not taken into account and the overall situation cannot be fully reflected in the hybrid model, a spatiotemporal hybrid model is proposed. The measured values and coordinates of all the typical points with pendulums of the arch dam are included in one spatiotemporal hybrid model, which is feasible, convenient, and accurate. The model can predict the deformation of any position on the arch dam. This is of great significance for real-time monitoring of deformation and stability of Jinping-I arch dam and ensuring its operation safety.

## 1. Introduction

In order to understand the operation state of Jinping-I arch dam, a large number of monitoring points are set up. The monitoring data of dam environmental quantity, such as deformation and temperature, is acquired regularly, so as to build the dam monitoring system. As an important feature, deformation data analysis is particularly important [1,2,3,4,5]. In fact, the deformation data of arch dam comes from the monitoring points with different spatial coordinates. Then the data not only has the time property, but also has spatial property, which is typical spatiotemporal data. In addition, because of the integrity of the dam structure in different degrees, the operational behaviors of the adjacent monitoring points affect and correlate with each other. At present, a lot of analysis is carried out on the characteristics of dam deformation on time [6,7,8], but there is little research on its time–space characteristics.

More and more attention has been paid to the representation methods which are specially aimed at the characteristics and requirements of spatiotemporal data [9,10,11,12,13,14,15], such as panel data, spatial panel data, and other emerging methods. Many scholars have conducted a lot of research in this regard. As a kind of typical spatiotemporal data [16,17,18,19,20], the deformation of Jinping-I arch dam is characterized by time, space, complexity, and uncertainty. In order to improve the quality of the spatiotemporal data mined in Jinping-I arch dam, it is necessary to preprocess the polluted spatiotemporal data.

According to the prototype monitoring data, the dam safety monitoring model is established by means of mathematics, mechanics, and information science [21]. The main task of the model is to establish the mathematical monitoring model based on the measured deformation data and monitor the arch dam operation status.

According to the different methods, the safety monitoring model can be summarized as: statistical model, grey system model, fuzzy mathematical model, etc. [22,23,24,25]. The basic characteristic of these models is to use the measured data, take the monitoring deformation as random variable, and apply the mentioned methods to establish various mathematical models. In essence, they are empirical models. The following problems may exist: (1) When the monitoring data does not include extreme load or the data series are short, the mathematical models established by these data will be difficult to be used for monitoring; (2) These models mainly rely on mathematical processing and the structural state of the dam and the dam foundation are not connected with this. Therefore, the dam working state cannot be explained essentially from the mechanical concept. However, the above problems can be better solved in the hybrid model [26].

In general, in view of the above data processing and safety monitoring model, this paper will do the following research:

(1) Taking Jinping-I arch dam as the object, this paper explores the data representation methods, which are suitable for monitoring deformation spatiotemporal data analysis, analyzes the category and source of monitoring deformation spatiotemporal data pollution, and studies the processing method of data missing [27], so as to improve the data quality.
(2) Combined with the actual working behavior of the dam and dam foundation, aiming at the clear relationship between the water depth and the dam deformation, the finite element method is used to calculate the effect field (such as displacement field and stress field) under the action of water pressure load [28], and the deterministic relationship between the water depth and dam deformation is established. Then, other components are still obtained by a statistical model [29]. The model is optimized and fitted with the measured data to obtain the adjustment parameters so as to establish the dam deformation safety monitoring hybrid prediction model [30,31,32,33,34].
(3) The hybrid model of single measuring point does not consider the relationship among measuring points, which cannot fully reflect the overall situation. Also, there will be too many hybrid models for each measuring point. It is difficult to predict the deformation of dam position without measuring points. Therefore, this paper establishes the deformation spatiotemporal hybrid model [35,36], in which the multiple measuring points in space are used and the spatial coordinate variables of points are introduced. One spatiotemporal hybrid model is used to estimate the deformation of the dam at any position by including the measured values of all typical measuring points.

The research is of great significance for real-time monitoring of deformation and stability of a concrete arch dam and ensuring its operation safety [37,38,39,40].

## 2. Deformation Data Analysis of Jinping–I Arch Dam

### 2.1. Deformation Data Representation Methods

As one of the important indexes of monitoring data for Jinping-I arch dam, the representation method of deformation data is developing and evolving. In the following, several data representation methods are discussed.

(1) Time series representation

**Figure 1:**
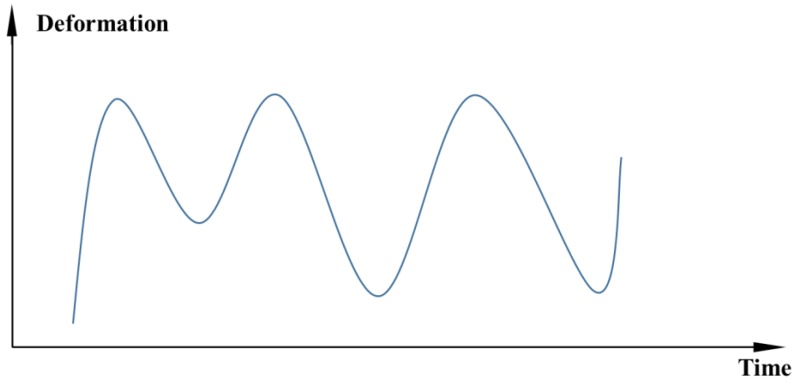
Time series data diagram.

Time series representation is the most common method in the traditional dam deformation analysis. Generally, for a certain measuring point in the monitoring system, the deformation value and the acquisition time point correspond one by one. Typical time series data is shown in Figure 1.

The data structure of time series is shown in Table 1.

The data structure of time series is relatively simple, which can be very concise to show the process of deformation value changing with time of measuring points. However, for each measuring point, if a separate time series is established, the data series will be relatively complex. At the same time, because of the simplicity of data structure, the information available for analysis is relatively limited, and the processing ability for missing variable deviation is not strong.

(2) Cross-section data representation

**Figure 2:**
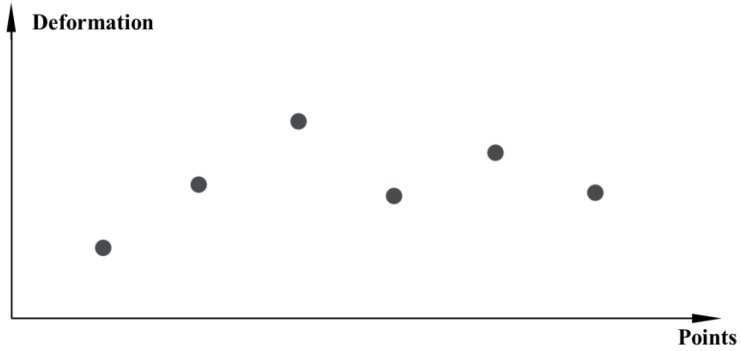
Cross-section data diagram.

In some specific cases, it is necessary to analyze the deformation of different monitoring points at a certain time point of the arch dam. For example, the overall deformation of the arch dam on a certain date and the distribution of the deformation value of the measuring point on the cross section, so as to monitor the abnormal point. At a specific time point, the deformation data formed by all the measuring points is called cross-section data. Typical cross-section data is shown in Figure 2.

It is assumed that there are n deformation monitoring points in an arch dam. For a specific time point, the structure of its cross-section data is shown in Table 2.

Cross-section data can only reflect the deformation value of the measuring point at a certain time, which cannot reflect the change of deformation value with time. In addition, the time series length of different measuring points is often different, so it is time-consuming, laborious, and inefficient to investigate the cross-section data of a certain time point.

(3) Panel data representation

**Figure 3:**
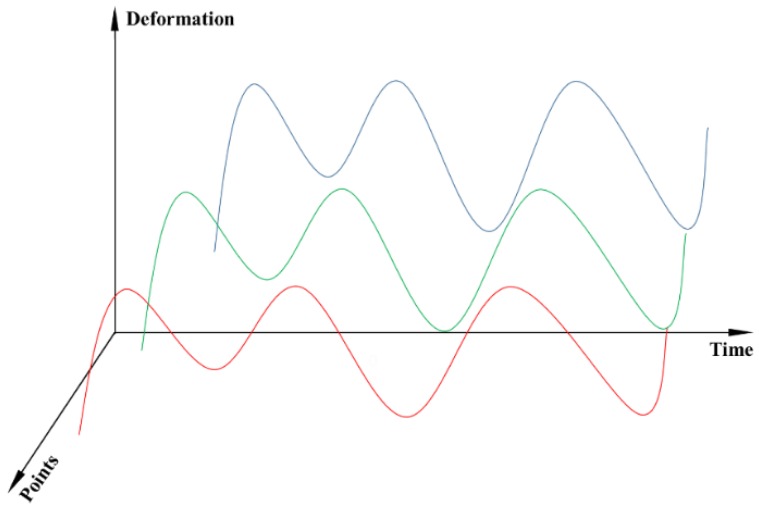
Panel data diagram.

Panel data refers to the data sequence of the same group of individuals over a period of time. Compared with the traditional time series, panel data adds cross-sectional dimension besides the time dimension. Therefore, it is named a two-dimensional data expression mode, which can express the deformation series of all measuring points at the same time. Typical panel data is shown in Figure 3.

Assuming that there are n deformation monitoring points and t time points in an arch dam, the panel data structure is shown in Table 3.

Obviously, panel data has two dimensions with n and t. Panel data is composed of time series of a group of measuring points, which combines time series and cross-section data at the same time. The main advantages are as follows. (1) Compared with time series, it can better solve the problem of missing variable deviation. (2) It has two dimensions of time series and cross-section at the same time, which can provide more dynamic behavior information of individuals and more accurate estimation.

(4) Spatial panel data representation

For the deformation monitoring data of Jinping-I arch dam, the location of the monitoring points is also concerned, so as to determine the abnormal location. In addition, the deformation of the adjacent measuring points in space may also affect each other. On the basis of the original panel data, the spatial coordinates (or spatial relations) of each measuring point is further considered. It is equivalent to adding the spatial coordinates of different points to the cross-section data in the two-dimensional panel data (time and cross-section). The data structure of the spatial panel is shown in Table 4.

**Figure 4:**
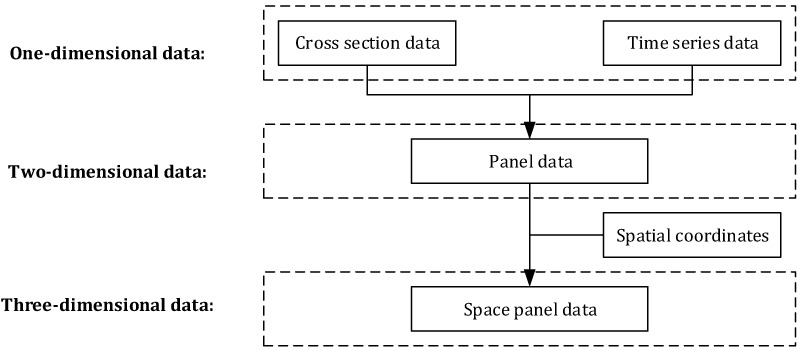
Relationships of several data representation methods.

From time series, cross-section data and panel data to spatial panel data, it is easy to find that the dimension of data and the capacity of samples are growing, and the information available for mining is also increasing geometrically with the change of data representation methods. The relationship of the several data representation methods is shown in Figure 4.

Spatial panel data considers the spatial coordinates of different measuring points, which facilitates the study of spatial relations of them. At the same time, the amount of information contained in spatial panel data is far larger than the traditional data representation method, which is an ideal mining object for spatiotemporal data analysis. Therefore, the content of spatiotemporal data analysis in this paper is based on spatial panel data.

### 2.2. Preprocessing of Spatiotemporal Deformation Data

The preprocessing of spatiotemporal deformation data is the premise of spatiotemporal data analysis. Spatiotemporal data analysis involves a large amount of deformation data from multiple monitoring points, most of which are polluted to varying degrees, resulting in a variety of errors and anomalies. At the same time, some deformation data is redundant, completely unrelated, or lost, which may interfere with the discovery of valuable spatiotemporal rules or patterns. This section will introduce some pretreatment methods existing in the deformation data.

Deformation data is affected by many factors in the process of obtaining, so the problem of data pollution is also diverse. The spatiotemporal data pollution is an objective phenomenon, which cannot be completely avoided, and can only be reduced by certain means. For the dam that has been built, the pretreatment can be started from the following aspects:

(1) During the dam operation, it is necessary to ensure the instruments maintenance to prevent the loss or error of deformation data. For the key monitoring location, the combination of manual monitoring and instrument monitoring is supposed to be adopted to ensure the authenticity and integrity of the data. For the newly found abnormal deformation location, deformation monitoring instruments is necessary to be added to track the deformation data in time.
(2) In the analysis stage of deformation data, only by fully extracting the influencing factors of dam deformation and establishing a relatively complete and reasonable deformation analysis model can the deformation state of dam be truly reflected to a greater extent.

In practical engineering, due to uncontrollable reasons such as human error and instrument damage, deformation data are often missing. For the missing spatiotemporal data, there are two main processing methods at present.

(1) Neglect method. In general, the real deformation data cannot be recovered completely, the unreasonable estimation of the missing value may lead to errors in the analysis model, so the missing data is neglected and not processed. To some extent, this method is reasonable and common.
(2) Likelihood method. In some specific cases, if it is necessary to study the spatial distribution of cross-section data at a certain time, it is still necessary to estimate the missing data and replace the missing value with the most likely likelihood value at that time.

As a typical large-scale spatial structure, Jinping-I arch dam has certain integrity in different scales. Thus, its deformation value distribution has certain continuity in space. Based on this assumption of spatial continuity, this paper proposes a method to estimate some missing deformation values.

(1) Interpolation of spatial neighboring points

**Figure 5:**
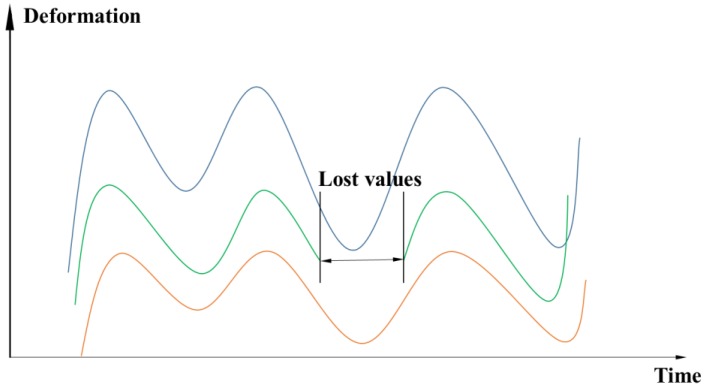
Partial data missing diagram.

It is assumed that there are three monitoring points, A, B, and C, which are close to each other in space and related to the structure of an arch dam. Among them, the data of measuring points A and C are complete, but one section of data of measuring point B is missing, as shown in Figure 5.

Considering the spatial proximity of measuring points A, B, and C, the missing value of measuring point B should be related to that of measuring point A and C in the missing period. Therefore, the deformation sequence of measuring point B can be expressed as a function of the deformation value of measuring point A and C. The expression is as follows:

(1) $$\delta_{B}=g\left(\delta_{A}\right)+g\left(\delta_{C}\right)+\varepsilon$$

where $g\left(\delta_{A}\right)$ and $g\left(\delta_{C}\right)$, respectively, represent the functions related to the deformation value of the measuring point A and C. They can be expressed by polynomials. Then,

(2) $$\delta_{B}=\alpha_{A}\overset{K_{A}}{\underset{i=0}{\sum }}\lambda_{A_{i}}\delta_{A}^{i}+\alpha_{C}\overset{K_{C}}{\underset{i=0}{\sum }}\lambda_{C_{i}}\delta_{C}^{i}+\beta_{B}+\varepsilon$$

where $\alpha_{A}$ and $\alpha_{C}$ are coefficients of polynomials containing $\delta_{A}$ and $\delta_{C}$ respectively; $K_{A}$ and $K_{C}$ are the highest degree of polynomials, which can be determined by drawing the scatter diagram of correlation between $\delta_{A}$, δc and $\delta_{B}$; $\lambda_{A_{i}}$ and $\lambda_{C_{i}}$ are weight coefficients respectively; $\beta_{B}$ is the translation term; ε is the mean error.

Furthermore, if considering the more general situation, for the deformation series of any monitoring point with missing data, it can be estimated as:

(3) $$\delta_{it}=\overset{L}{\underset{j=1}{\sum }}\alpha_{ij}g\left(\delta_{jt}\right)+\beta_{i}+\varepsilon$$

where L represents the number of measurement points close to measurement point i (a certain critical distance can be set to judge the proximity between different points); $\delta_{jt}$ represents the deformation series of measurement points close to $\delta_{it}$; $g(\delta_{jt})$ represents the correlation function between $\delta_{it}$ and $\delta_{jt}$; $\beta_{i}$ represents the translation term of measurement point.

According to the deformation data of $\delta_{it}$ and the adjacent measuring points, the least square method can be used to estimate the value of $\alpha_{ij}$, so as to determine the model expression finally.

(2) Space inverse distance weighted interpolation

If the least squares estimate is not effective or the lack of data is serious, the inverse distance weighted spatial interpolation method can be used to estimate the deformation value. That is to say, the data of missing measuring points is the result of weighted average of adjacent measuring points. At this time, the weight sum of deformation values of adjacent measuring points is 1, that is:

(4) $$\overset{L}{\underset{j=1}{\sum }}\alpha_{ij}=1$$

(5) $$\delta_{it}=\overset{K}{\underset{j=1}{\sum }}\alpha_{ij}\delta_{jt}+\varepsilon$$

where $\alpha_{ij}$ is related to the space distance between the measuring point i and the surrounding measuring point. According to ‘Geography First Law’, it is generally believed that the closer the space is, the stronger the correlation between the two points is. Therefore,

(6) $$\alpha_{ij}=\frac{{\left(d_{ij}\right)}^{-\gamma }}{\sum_{m=1}^{L}{\left(d_{ij}\right)}^{-\gamma }}$$

(7) $$d_{ij}=\sqrt{{\left(x_{i}-x_{j}\right)}^{2}+{\left(y_{i}-y_{j}\right)}^{2}+{\left(z_{i}-z_{j}\right)}^{2}}$$

where $\alpha_{ij}$ is the space distance between measuring point i and j; γ is generally taken as 1 or 2. Its advantage is that the estimated value of deformation is only related to the spatial position and the deformation value of any point can be estimated in a certain spatial range.

## 3. Spatiotemporal Hybrid Security Monitoring Model

According to the prototype monitoring data of the dam, the dam safety monitoring model is established with the application of mathematics, mechanics, information science, and other methods. The main task is to use the model to monitor the dam operation. The physical quantity obtained from deformation or stress monitoring is very important for monitoring the operation conditions. The deformation is intuitive and reliable, which is generally regarded as the most important monitoring quantity at home and abroad. In order to master the real operation state of the dam, it is necessary to analyze the measured data of each deformation and establish the prediction equation.

According to the different methods of model building, the models can be summarized as: statistical model, grey system model, fuzzy mathematical model, analysis and calculation with finite element method, hybrid model, etc.

Considering the lack of these models above, which is introduced in the introduction above, this paper selects the hybrid model as the monitoring method for the dam deformation.

### 3.1. Principle of Hybrid Model

The deformation of Jinping-I arch dam is a comprehensive reflection of several environmental factors. The relationships between some environmental factors (such as the water depth in front of the dam) and the deformation are clear. Through the corresponding mathematical and mechanical methods, the relationship between them can be established. When the relationship is not clear or it is difficult to be established with theoretical analysis, the statistical analysis and structural calculation will be used. Therefore, the hybrid model of dam deformation safety monitoring can be constructed.

Under the external load (water pressure, temperature, etc.), the deformation at any point can be divided into hydraulic component, temperature component, and time effect component according to their causes.

(8) $$\delta =\delta_{H}\left(t\right)+\delta_{T}\left(t\right)+\delta_{\theta }\left(t\right)$$

where δ is the measured deformation value; $\delta_{H}\left(t\right),\;\delta_{T}\left(t\right)$, and $\delta_{\theta }\left(t\right)$ are hydraulic component, temperature component, and time effect component, respectively.

(1) Hydraulic component

The hydraulic component $\delta_{H}\left(t\right)$ is calculated by finite element method. According to the known mechanical parameters of the dam body and dam foundation, the finite element method is used to calculate the deformation at each point $\delta_{H}{}_{1},\delta_{H}{}_{2}\cdots ,\delta_{H}{}_{n}$ under different water depth $H_{1},H_{2}\cdots ,H_{n}.\;$

(9) $$\delta_{H}=\overset{4}{\underset{i=1}{\sum }}a_{i}\left(H^{i}-H_{0}^{i}\right)$$

where $a_{i}$ is the regression coefficient; H is the water depth in front of the dam during deformation observation, which is the reservoir water level minus the of the dam bottom elevation.

(2) Temperature component

When the embedded thermometer of the dam body is insufficient, the internal temperature of the dam body reaches the quasi stable temperature field. Generally, temperature loading is taken as a periodic (harmonic) function, which can basically meet the modeling requirements. The harmonic factor is selected to approximate the change of temperature field of Jinping-I arch dam. That is to say:

(10) $$\delta_{T}=\overset{2}{\underset{i=1}{\sum }}[b_{1i}\left(\sin\frac{\;2\pi it}{365}-\sin\frac{\;2\pi it_{0}}{365}\right)+b_{2i}\left(\cos\frac{\;2\pi it}{365}-\cos\frac{\;2\pi it_{0}}{365}\right)$$

where t is the accumulated days from the monitoring date to the starting monitoring date; $t_{0}$ is accumulated days from the starting date of the data series to the first monitoring date; $b_{1i}$ and $b_{2i}$ are the regression coefficients of temperature component.

(3) Time effect component

The reason of time effect deformation of Jinping-I dam is very complex, such as the creep and plastic deformation of the concrete, irreversible deformation caused by dam cracks and autogenous volume deformation. For normal operation dams, the change rule of time effect deformation is that the initial change is sharp, and the later change is gradually stable. The time effect component of displacement change during the normal operation can be expressed as:

(11) $$\delta_{\theta }=c_{1}\left(\theta -\theta_{0}\right)+c_{2}\left(ln\theta -ln\theta_{0}\right)\;$$

where $\theta =t/100,\theta_{0}=t_{0}/100$; $c_{1}$ and $c_{2}$ are the regression coefficients.

In conclusion, considering the influence of the initial value, the tracking prediction and analysis hybrid model of Jinping-I dam deformation is obtained as follows:

(12) $$\delta =\overset{4}{\underset{i=1}{\sum }}a_{i}\left(H^{i}-H_{0}^{i}\right)\;+\overset{2}{\underset{i=1}{\sum }}\left[b_{1i}\left(\sin\frac{\;2\pi it}{365}-\sin\frac{\;2\pi it_{0}}{365}\right)+b_{2i}\left(\cos\frac{\;2\pi it}{365}-\cos\frac{\;2\pi it_{0}}{365}\right)\right]+c_{1}\left(\theta -\theta_{0}\right)+c_{2}\left(ln\theta -ln\theta_{0}\right)$$

where $a_{0}$ is a constant term, and other symbols have the same meanings in Equation (8) to Equation (11).

In this paper, the calculating deformation of the established model is evaluated with the residual standard deviation (S) and the correlation coefficient (R).

(13) $$S=\sqrt{\frac{1}{n}\overset{n}{\underset{t=1}{\sum }}{\left(\delta_{t}^{M}-\delta_{t}^{C}\right)}^{2}}$$

(14) $$R=\sqrt{\overset{n}{\underset{t=1}{\sum }}{\left(\delta_{t}^{c}-\frac{1}{n}\overset{n}{\underset{t=1}{\sum }}\delta_{t}^{M}\right)}^{2}/\overset{n}{\underset{t=1}{\sum }}{\left(\delta_{t}^{M}-\frac{1}{n}\overset{n}{\underset{t=1}{\sum }}\delta_{t}^{M}\right)}^{2}}$$

where $\delta_{t}^{M}$ and $\delta_{t}^{c}$ are the measured and calculated values of deformation, respectively; n is the number of the measured values.

### 3.2. Spatiotemporal Hybrid Model

The above-introduced is a single point model, which does not consider the spatial relationship among the measurement points. At the same time, the single point model does not fully reflect the overall situation, which will affect the analysis accuracy. Also, there will be too many hybrid models for each point. It is difficult to predict the deformation of dam position without measuring points. In view of the above problems, this paper puts forward a deformation spatiotemporal hybrid model, in which the multiple measuring points in space are used and the spatial coordinate variables of points are introduced.

(15) δ=f(H,T,θ,x,y,z)

where H is the hydraulic factor; T is the temperature factor; θ is the time effect factor caused by the creep of concrete and the rheology of bedrock; x, y, and z are the the spatial coordinate variables.

The spatiotemporal hybrid model can timely understand the displacement field of dam body under any load combination (H,T,θ,⋯) at a certain time and master the situation that the displacement of the location (x,y,z) deviates from the real displacement field due to local factors. Then, we can find out the abnormalities as early as possible, analyze the causes, take countermeasures, and eliminate hidden dangers. At the same time, when a certain coordinate (x,y,z) is fixed, the above model is the hybrid model of a specific measuring point. In addition, using the above model for analysis, the results can reflect the overall situation of the dam due to the connection of multiple measuring points. The above-mentioned methods and theories have been successfully applied to Danjiangkou, Longyangxia, Foziling, Three Gorges, and other dams. The following describes the principle and calculation formula of the spatial-temporal hybrid model.

It can be seen from the previous analysis that under the action of water pressure and temperature and considering the creep of dam concrete and the rheology of bedrock, the displacement field of dam and dam foundation will be generated.

(1) Calculation principle and formula of each component

As we all know, in the small deformation range, under the action of external loads (water pressure, temperature, etc.), the deformation at any point of the dam and dam foundation can be divided into hydraulic component, temperature component, and time effect component according to their causes, as shown in Equation (15). It will not be described in detail here. Considering the spatial distribution, the spatiotemporal hybrid model is established as follows:

(16) $$\delta =f\left(H,T,\theta ,x,y,z\right)=f_{1}\left(H,x,y,z\right)+f_{2}\left(T,x,y,z\right)+f_{3}\left(\theta ,x,y,z\right)=f_{1}\left[f\left(H\right),f\left(x,y,z\right)\right]+f_{2}\left[f\left(T\right),x,y,z\right]+f_{3}\left[f\left(\theta \right),f\left(x,y,z\right)\right]$$

Combined with Equation (12), spatiotemporal hybrid model is obtained as

(17) $$\delta =\overset{3\left(4\right)}{\underset{k=0}{\sum }}\overset{3}{\underset{l,m,n=0}{\sum }}A_{klmn}H^{k}x^{l}y^{m}z^{n}+\overset{1}{\underset{j,k=0}{\sum }}\overset{3}{\underset{l,m,n}{\sum }}B_{jklmn}\frac{\sin2\pi jt}{365}\cdot \frac{\cos2\pi kt}{365}x^{l}y^{m}z^{n}+\overset{1}{\underset{j,k=0}{\sum }}\overset{3}{\underset{l,m,n=0}{\sum }}C_{jklmn}\theta_{j}\cdot ln\theta_{k}x^{l}y^{m}z^{n}$$

When the deflection curve of a beam is studied, the above model degenerates into:

(18) $$\delta =\overset{3\left(4\right)}{\underset{k=0}{\sum }}\overset{3}{\underset{n=0}{\sum }}A_{kn}H^{k}z^{n}+\overset{1}{\underset{j,k=0}{\sum }}\overset{3}{\underset{n}{\sum }}B_{jkn}\frac{\sin2\pi jt}{365}\cdot \frac{\cos2\pi kt}{365}z^{n}+\overset{1}{\underset{j,k=0}{\sum }}\overset{3}{\underset{n=0}{\sum }}C_{jkn}\theta_{j}\cdot ln\theta_{k}z^{n}$$

When a horizontal arch is studied, the above model degenerates into:

(19) $$\delta =\overset{3\left(4\right)}{\underset{k=0}{\sum }}\overset{3}{\underset{l,m=0}{\sum }}A_{klmn}H^{k}x^{l}y^{m}+\overset{1}{\underset{j,k=0}{\sum }}\overset{3}{\underset{l,m=0}{\sum }}B_{jklm}\frac{\sin2\pi jt}{365}\cdot \frac{\cos2\pi kt}{365}x^{l}y^{m}+\overset{1}{\underset{j,k=0}{\sum }}\overset{3}{\underset{l,m=0}{\sum }}C_{jklmn}\theta_{j}\cdot ln\theta_{k}x^{l}y^{m}$$

(2) Estimation of parameters $\left(A_{i},B_{i},C_{i}\right)$ in the model

The coordinates (x,y,z) of each measuring point and the water depth H, temperature T, and time effect θ corresponding to the measured deformation $\delta^{M}\left(H,t,\theta ,x,y,z\right)$ are substituted into the above equations. The least square method is used for optimal fitting, so as to obtain the parameters in the above model. Namely

(20) $$Q=\sum^{}{\left[\delta^{M}\left(H,T,\theta ,x,y,z\right)-f\left(H,T,\theta ,x,y,z\right)\right]}^{2}$$

Take the partial derivative of Equation (20), and get:

(21) $$\frac{\partial Q}{\partial A_{i}}=0,\;\frac{\partial Q}{\partial B_{i}}=0,\;\frac{\partial Q}{\partial C_{i}}=0$$

Then the parameters $\left(A_{i},B_{i},C_{i}\right)$ in the model are obtained, that is, the spatial-temporal distribution model of spatial displacement field is established.

## 4. Case Study

### 4.1. Project Overview

Jinping-I hydropower station is the key project of the Yalong River, which is located in Sichuan Province. The project is mainly for power generation and also for flood control. The normal water level of the reservoir is 1880 m, the dead water level is 1800 m, the storage capacity below the normal water level is 7.76 billion m^3^, and the regulating storage capacity is 4.91 billion m^3^.

**Figure 6:**
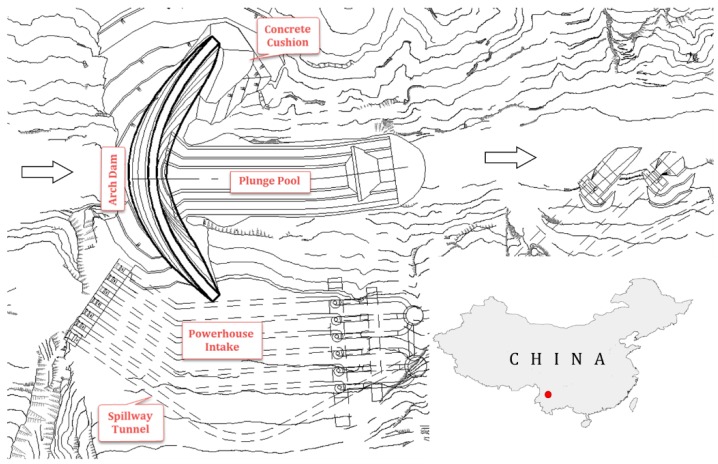
Layout chart of Jinping-I arch dam.

The main hydraulic structures of Jinping-I hydropower station are composed of concrete arch dam, cushion, plunge pool, powerhouse intake and spillway tunnel. Figure 6 is the layout chart of the Jinping-I hydropower station. The dam crest elevation is 1885.0 m, with the maximum dam height of 305.0 m.

**Figure 7:**
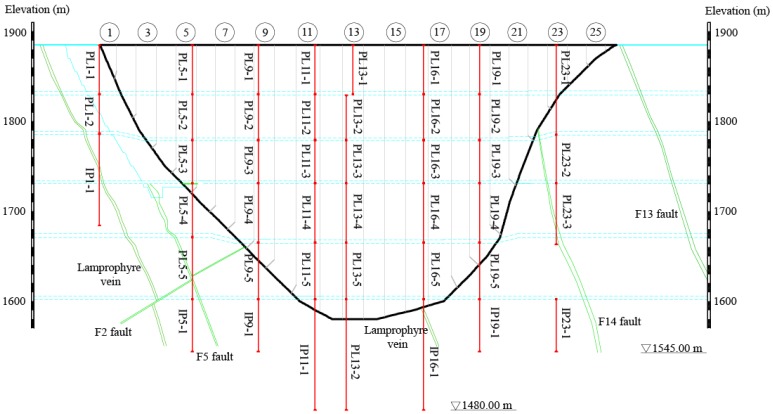
Layout of the pendulum system of Jinping-I arch dam.

Jinping-I arch dam has a large scale and a high level of main buildings. Dam horizontal displacements are measured by plumb lines. The specific layout of the pendulum system is shown in Figure 7. For dam monitoring displacement, the general rule is: radial displacement is positive to downstream, and tangential displacement is positive to left bank.

Figure 7 shows that the distribution of pendulum system is roughly uniform along the height direction, the same in the left and right bank direction, which can reflect the deformation state of the dam comprehensively. Figure 7 shows the rationality and effectiveness of the pendulum system.

### 4.2. Deformation Data Representation

**Figure 8:**
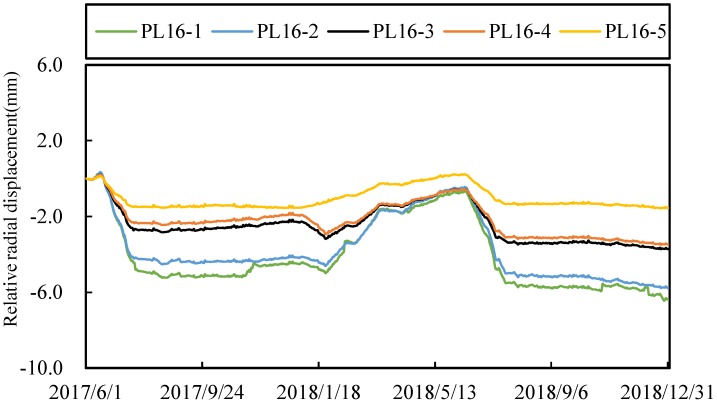
Relative radial displacement distribution of PL16-1~PL16-5.

In order to show how to use the spatial panel data representation method to express the deformation data, radial displacements of No. 16 dam section are taken as an example. The relative radial displacement distribution of PL16-1~PL16-5 measuring points on the dam section is shown in Figure 8 from 1 June 2017 to 30 December 2018. It can be found that the deformation process lines of these measuring points have a certain correlation.

**Figure 9:**
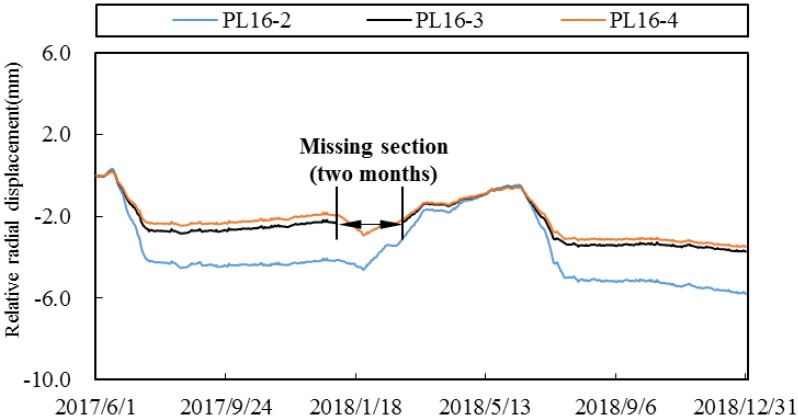
Deformation missing data process lines.

In view of the possible pollution problem in the deformation data, it is necessary to preprocess the spatiotemporal data. Here, we mainly discuss the estimation problem of the deformation missing data. In order to verify the feasibility of the method in Section 2.2, taking the measuring point PL16-3 in Figure 8 as an example, the missing section for up to two months (January and February 2018) are artificially constructed, as shown in Figure 9.

**Figure 10:**
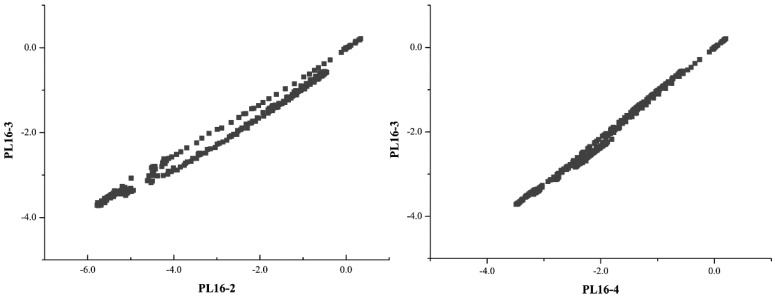
Distribution diagram of correlation between measuring points.

The correlation between PL16-3 and other two measuring points PL16-2 and PL16-4 is expressed in the form of correlation diagram, respectively, as shown in Figure 10.

According to the results of Figure 10, the correlation between the measuring point PL16-3 and the adjacent measuring point is obviously linear. For the missing section estimation, the estimated formulas based on spatial proximal point interpolation and spatial inverse distance interpolation are:

(22) $$\delta_{PL16-3}^{C}=0.193\delta_{PL16-2}+0.746\delta_{PL16-4}-0.070$$

(23) $$\delta_{PL16-3}^{C}=0.206\delta_{PL16-2}+0.794\delta_{PL16-4}$$

**Figure 11:**
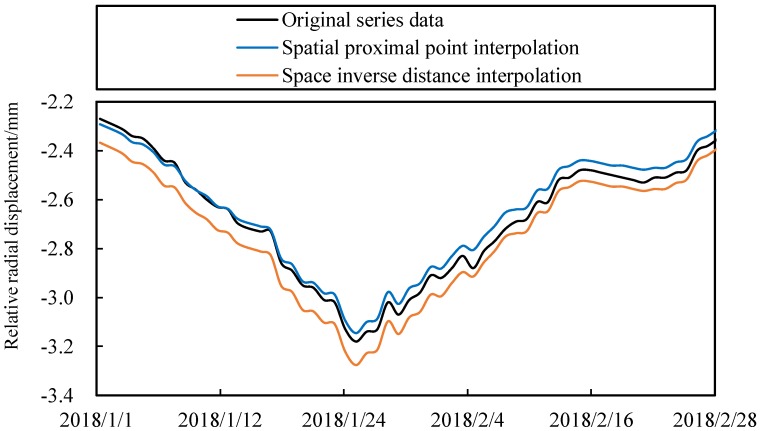
Estimation of deformation missing data process lines.

According to Figure 11 and Table 5, it can be found that the missing value estimation method proposed in this paper has high accuracy. The estimation effect of spatial neighbor interpolation method is better. However, the advantage of spatial inverse distance weighted interpolation method is that it can estimate the deformation value of any measuring point in a certain space.

### 4.3. Hybrid Model Analysis

#### 4.3.1. Finite Element Model of Jinping-I Arch Dam

In this paper, the hybrid model of dam horizontal displacement is established by combining the finite element numerical simulation method with the statistical method, so as to ensure the long-term operation of Jinping-I arch dam.

**Figure 12:**
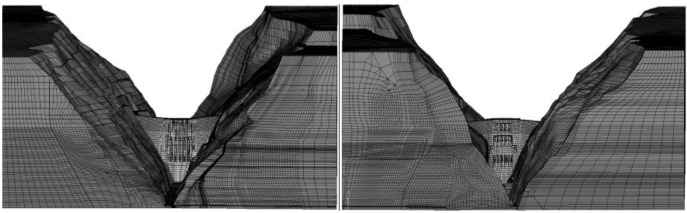
Three-dimensional finite element model of Jingping-I arch dam (upstream and downstream viewpoints).

**Figure 13:**
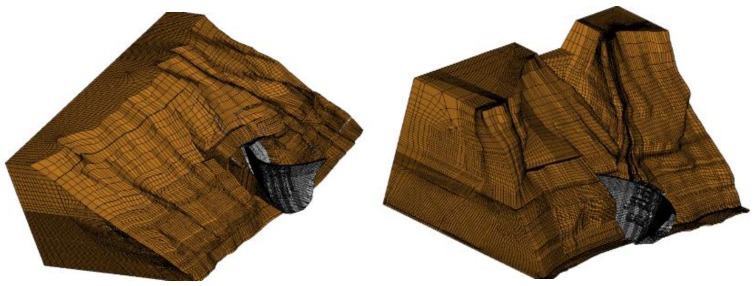
Three-dimensional finite element simulation of left and right banks.

**Figure 14:**
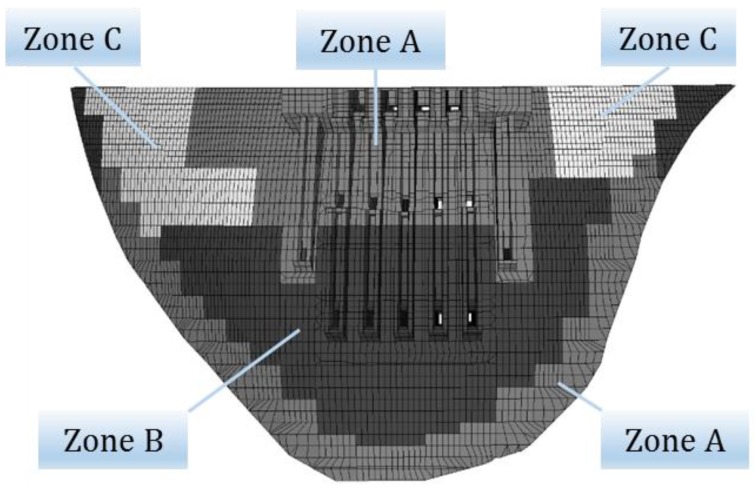
The finite element model of zoning concrete of dam body.

Based on engineering design and geological data, a three-dimensional finite element model of Jinping-I arch dam is established to study dam working state. The finite element is built according to the two-dimensional engineering drawings and the simulation calculation is conducted with the finite element software. Figure 12 shows the finite element model which consists of 923,737 elements and 957,221 nodes, and the number of nodes and elements for the dam body is 36,079 and 29,840, respectively. The mesh for dam body and foundation are relatively fine and coarse by progressive meshing technique to achieve a balance between accuracy and efficiency of the simulation. Furthermore, several models with different element sizes were established for mesh validation, which demonstrated that the selected model satisfy the demand calculation accuracy. Figure 13 simulates the shape of the mountain. Figure 14 shows the zoning concrete of dam body model. The arch dam body is divided into zone A, zone B, and zone C, according to the construction manual.

Combined with the concrete test results, the modulus of elasticity for main zones are shown in Table 6.

#### 4.3.2. Calculation Results of the Hybrid Model

**Figure 15:**
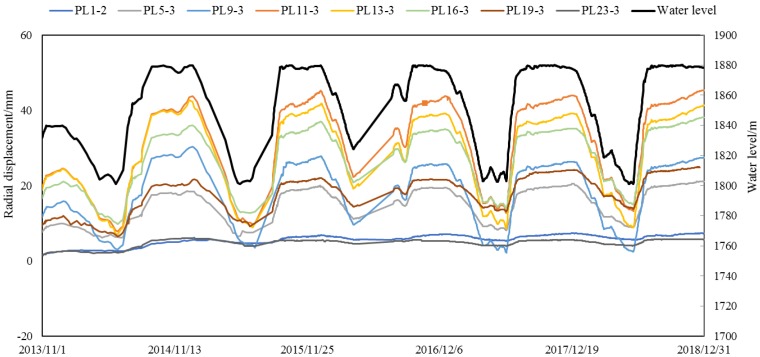
Time series of several radial displacement of some dam sections and water level.

According to the radial displacement data of Jinping-I arch dam from 1 November 2013 to 31 December 2018, a hybrid model is established. The hydraulic component is determined according to the finite element model on different water level, and the temperature component and time effect component are determined by the statistical model. Figure 15 shows time series of several radial displacement of some dam sections and water level. We can see that the deformation is greatly affected by the change of water level. Therefore, in the FEM model analysis, water level is an important factor to be considered.

In the following, 42 typical measuring points on No. 1, No. 5, No. 9, No. 11, No. 13, No. 16, No. 19, and No. 23 dam sections are used to establish the hybrid model. From 1 November 2013 to 31 December 2018, the upstream water level mainly changed between 1700 m and 1880 m. In the process of determining the hydraulic component in the finite element model, the upstream water level is selected every 5 m between 1700 m and 1880 m. A total of 37 sets of hydraulic loads are calculated by finite element method.

According to the specific situation of Jinping-I arch dam, the finite element structural analysis software ABAQUS is used for analysis and calculation. Temperature component and time effect component are not considered temporarily.

According to the finite element calculation, the radial displacement of 42 typical measuring points of the dam body under the load of 37 groups of upstream water level from 1700 m to 1880 m can be obtained. The regression trend line can be fitted, and the functional relationship between the radial displacement and the upstream water level can be obtained as the calculation basis of the hydraulic component in the hybrid model.

(24) $$\delta_{H}=a_{0}+a_{1}H+a_{2}H^{2}+a_{3}H^{3}+a_{4}H^{4}$$

Here, measuring point PL13-2 is selected to briefly explain the calculation process. Table 7 is the finite element calculation table of hydraulic component of measuring point PL13-2.

In order to reduce the error of fitting function, the upstream water depth is firstly normalized.

(25) $$H=\frac{H_{u}-H_{b}}{H_{d}}$$

where $\;H_{b}=1580$, which is dam base elevation and $H_{d}$ = 305, which is the maximum dam height.

**Figure 16:**
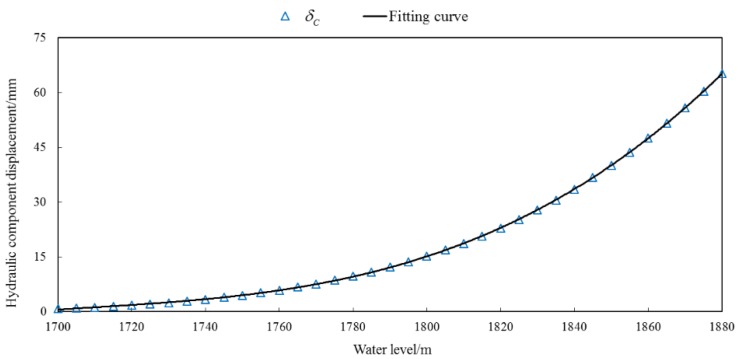
Fitting curve between hydraulic component displacement calculated by FEM of PL13-2 and water level.

According to the relationship between the displacement calculated by the finite element method and the normalized water level  H, the fitting curve is fitted, which is shown in Figure 16. The expression is as follows:

(26) $$\delta_{H}=-11.588-67.402\;H-128.580H^{2}-59.787H^{3}+83.438H^{4}$$

According to the Equation (26), the regression fitting displacement can be obtained and used as the calculation basis of the hydraulic component of PL13-2 in the hybrid model.

The coefficients of the relationship curve between the calculated displacement of finite element and the normalized water level H of all typical measuring points can be obtained.

After the function relationship between the displacement calculated by FEM and the normalized water level H  of each typical measuring point is obtained, the hydraulic component can be calculated according to the measured water level as the hydraulic component factor in the hybrid model, and then the temperature component and the time effect component can be calculated. Therefore, the hybrid model can be established. According to the relationship between the fitting hydraulic component and water level, substituting Equation (24) into Equation (12), we can get:

(27) $$\delta =a_{0}+a\delta_{H}+\overset{2}{\underset{i=1}{\sum }}\left[b_{1i}\left(\sin\frac{\;2\pi it}{365}-\sin\frac{\;2\pi it_{0}}{365}\right)+b_{2i}\left(\cos\frac{\;2\pi it}{365}-\cos\frac{\;2\pi it_{0}}{365}\right)\right]+c_{1}\left(\theta -\theta_{0}\right)+c_{2}\left(ln\theta -ln\theta_{0}\right)\;$$

where the meaning of each parameter is the same as the above.

Table 7 shows the correlation coefficient R and residual standard deviation S of the hybrid model of the typical measuring points of the pendulums of the dam body. The accuracy of the model is high.

Table 8 shows the coefficients of the hydraulic component, temperature component, time effect component, and constant term of the hybrid model, that is, the coefficients in Equation (27).

**Figure 17:**
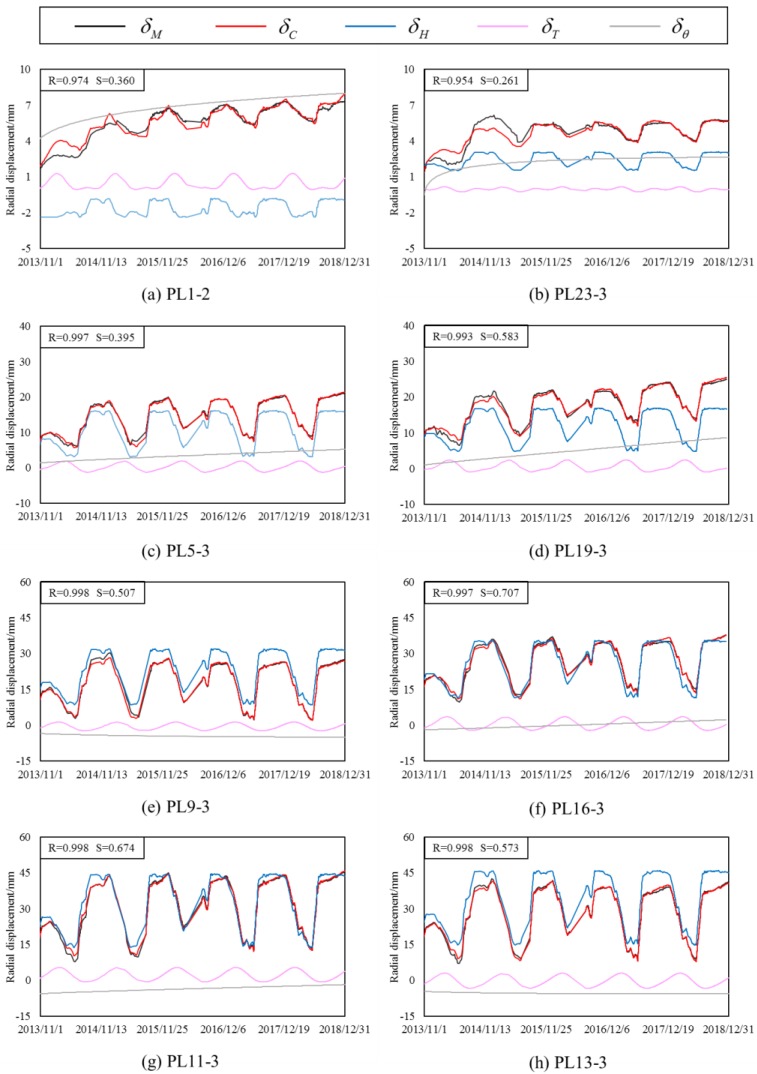
Fitting results of radial displacement hybrid model and component separation results of some dam sections.

According to the results of the hybrid model, the deformation of Jinping-I arch dam during high water level impoundment was separated from the hydraulic component, temperature component, and time effect component. In this paper, the fitting value and separation amount of radial displacement hybrid model of all typical measuring points are calculated. Due to space limitation, the results of some typical measuring points of dam section are shown in Figure 17. Figure 17 shows that the hybrid model established in this paper has reasonably high accuracy, and the radial displacement of symmetrical dam sections on the left and right banks is similar, which is in line with the actual situation.

### 4.4. Spatiotemporal Hybrid Model Results

The above-introduced is a single point model, which does not consider the spatial relationship among the measurement points. At the same time, the single point model does not fully reflect the overall situation, which will affect the analysis accuracy. Also, there will be too many hybrid models for each point. It is difficult to predict the deformation of dam position without measuring points. Therefore, combined with the relevant principles and contents of Equation (12) to Equation (20), this paper proposes a spatiotemporal hybrid model of Jinping-I arch dam.

Considering that the thickness of Jinping-I arch dam is relatively thin, the coordinate value of Y direction changes little relative to X direction or Z direction. Therefore, the influence of coordinate change of X direction and Z direction on radial deformation measurement in space are considered to establish the spatiotemporal hybrid model. Equation (27) is simplified as:

(28) $$\delta =a_{0}+\overset{3}{\underset{l,n=0}{\sum }}A_{ln}x^{l}z^{n}+\overset{1}{\underset{j,k=0}{\sum }}\overset{3}{\underset{ln}{\sum }}B_{jkln}\frac{\sin2\pi jt}{365}\cdot \frac{\cos2\pi kt}{365}x^{l}z^{n}+\overset{1}{\underset{j,k=0}{\sum }}\overset{3}{\underset{l,n=0}{\sum }}C_{jkln}\theta_{j}\cdot ln\theta_{k}x^{l}z^{n}$$

The spatiotemporal hybrid model established in this section includes the measured values of all the typical measuring points of pendulums of the dam body. That is, the spatiotemporal measured values of all the measuring points are taken into account in one specific spatiotemporal hybrid model.

Table 9 shows the spatial location of typical measuring points of Jinping-I arch dam based on the spatiotemporal hybrid model. After standardizing the X and Z coordinate values, we can substitute them into Equations (15) and (28) as (x,z).

(29) $$\delta =f\left(H,T,\theta ,x,z\right)=a_{0}+\overset{3}{\underset{l,n=0}{\sum }}A_{ln}x^{l}z^{n}$$

$$+\overset{1}{\underset{j,k=0}{\sum }}\overset{3}{\underset{ln}{\sum }}B_{jkln}\frac{\sin2\pi jt}{365}\cdot \frac{\cos2\pi kt}{365}x^{l}z^{n}+\overset{1}{\underset{j,k=0}{\sum }}\overset{3}{\underset{l,n=0}{\sum }}C_{jkln}\theta_{j}\cdot ln\theta_{k}x^{l}z^{n}$$

By the way, in this section, on the basis of the original single point hybrid model, the newly added x and z coordinates are fitted with cubic polynomials, respectively, so the parameters of the spatiotemporal hybrid model have a 4×4×7=112 terms.

**Figure 18:**
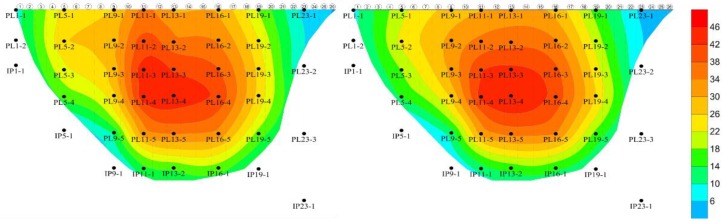
Comparison nephogram between the measured value (**Left**) and calculated value (**Right**) of spatiotemporal hybrid model of radial displacement of dam body on 31 December 2018 (unit: mm).

Table 10 shows the correlation coefficient and residual standard deviation of the spatiotemporal hybrid model of the typical pendulums of the dam body. The correlation coefficient shows that the fitting accuracy is good, while the residual standard deviation is larger than that of the single point hybrid model. However, considering that the measured values of the spatiotemporal hybrid model include all the measured points, the fitting accuracy of residual standard deviation is still high in the spatiotemporal model. Table 11 shows the spatiotemporal hybrid model coefficients in detail. Figure 18 shows comparison nephogram between the measured value and calculated value of radial displacement of dam body on 31 December 2018. It can be seen from Figure 18 that the fitting effect of the model is good.

To sum up, for all the typical measuring points in an arch dam, considering the factors of its spatial location, only a general spatiotemporal hybrid model is needed. The model is feasible and has high precision, which can basically be applied to the calculation of radial displacement of all positions on the dam body. It is of great significance for the safety monitoring of the arch dam.

## 5. Conclusions

In this paper, taking the deformation data of Jinping-I arch dam as an example, a spatiotemporal prediction model is established. The main research contents are summarized as follows:

(1) This paper studies the representation methods of monitoring deformation data, and analyzes the characteristics of various methods. We should choose the spatial panel data representation if possible, which is more suitable on deformation data analysis. Aiming at data pollution, this paper puts forward the regressions of interpolation of spatial neighboring points and the spatial inverse distance weighted interpolation methods, both of which are applicable. We can choose the methods when the missing data is important;
(2) Combined with the actual working behavior of Jinping-I arch dam, a hybrid model is established. The FEM is used to calculate the displacement field of the dam and its foundation under the action of hydraulic pressure. The statistical model is still used for the other components. The results show that the established hybrid model is feasible with high accuracy. During the dam operation, a hybrid model is necessary to be established to monitor the dam deformation at the measured points.
(3) Considering the lack of space influence in the single measuring point hybrid model, the spatiotemporal hybrid model of radial displacement is established by using multiple measuring points in space and introducing the spatial coordinate variables. The specific spatiotemporal hybrid model includes the measured values of all the typical measuring points of pendulums of the dam body. The spatiotemporal hybrid model is much easier after it is built. The model can be basically applied to the calculation of radial displacement at any position on the dam body. It can be proved that the established model is feasible, accurate, and applicable for the Jinping-I arch dam.

The methods mentioned above are applicable and of great significance for the safety monitoring of arch dams. However, in this paper, some extreme conditions (very cold winter or very warm summer) are not considered, which are worthy of further study.

## Figures and Tables

**Table 1:** Time series data structure.

Time	1	2	3	…	*t*
Deformation	$\;\delta_{1}\;$	$\;\delta_{2}\;$	$\;\delta_{3}\;$		$\;\delta_{t}\;$

**Table 2:** Cross-section data structure.

Points	1	2	3	…	*n*
Deformation	$\;\delta_{1}\;$	$\;\delta_{2}\;$	$\;\delta_{3}\;$		$\;\delta_{n}\;$

**Table 3:** Panel data structure.

Points	1	2	3	⋯	*t*
1	$\;\delta_{11}\;$	$\;\delta_{12}\;$	$\;\delta_{13}\;$	⋯	$\;\delta_{1t}\;$
2	$\;\delta_{21}\;$	$\;\delta_{22}\;$	$\;\delta_{23}\;$	⋯	$\;\delta_{2t}\;$
3	$\;\delta_{31}\;$	$\;\delta_{32}\;$	$\;\delta_{33}\;$	⋯	$\;\delta_{3t}\;$
⋮	⋮	⋮	⋮	⋱	⋮
n	$\;\delta_{n1}\;$	$\;\delta_{n2}\;$	$\;\delta_{n3}\;$	⋯	$\;\delta_{nt}\;$

**Table 4:** Space panel data structure.

Points	Space Coordinates	1	2	3	⋯	*t*
1	$\;\left(x_{1},y_{1},z_{1}\right)\;$	$\;\delta_{1}\left(x_{1},y_{1},z_{1}\right)\;$	$\;\delta_{2}\left(x_{1},y_{1},z_{1}\right)\;$	$\;\delta_{3}\left(x_{1},y_{1},z_{1}\right)\;$	⋯	$\;\delta_{t}\left(x_{1},y_{1},z_{1}\right)\;$
2	$\;\left(x_{2},y_{2},z_{2}\right)\;$	$\;\delta_{1}\left(x_{2},y_{2},z_{2}\right)\;$	$\;\delta_{2}\left(x_{2},y_{2},z_{2}\right)\;$	$\;\delta_{3}\left(x_{2},y_{2},z_{2}\right)\;$	⋯	$\;\delta_{t}\left(x_{2},y_{2},z_{2}\right)\;$
3	$\;\left(x_{3},y_{3},z_{3}\right)\;$	$\;\delta_{1}\left(x_{3},y_{3},z_{3}\right)\;$	$\;\delta_{2}\left(x_{3},y_{3},z_{3}\right)\;$	$\;\delta_{3}\left(x_{3},y_{3},z_{3}\right)\;$	⋯	$\;\delta_{t}\left(x_{3},y_{3},z_{3}\right)\;$
⋮	⋮	⋮	⋮	⋮	⋱	⋮
n	$\;\left(x_{n},y_{n},z_{n}\right)\;$	$\;\delta_{1}\left(x_{n},y_{n},z_{n}\right)\;$	$\;\delta_{2}\left(x_{n},y_{n},z_{n}\right)\;$	$\;\delta_{3}\left(x_{n},y_{n},z_{n}\right)\;$	⋯	$\;\delta_{t}\left(x_{n},y_{n},z_{n}\right)\;$

**Table 5:** Accuracy of the two estimation methods.

Method	Spatial Proximal Point Interpolation	Spatial Inverse Distance Interpolation
Coefficient of determination	0.998	0.987
Standard estimation error (mm)	0.046	0.053

**Table 6:** Modulus of elasticity for main zones of Jinping-I dam.

Locations	Volume (m^3^)	Modulus (GPa)
Zone A	2.23 ✕ 10^6^	30.7
Zone B	2.10 ✕ 10^6^	30.5
Zone C	5.97 ✕ 10^5^	28.0
Dam foundation	1.54 ✕ 10^9^	27.0

**Table 7:** Correlation coefficient and residual standard deviation of deformation hybrid model of typical measuring points.

Dam Section	Points	R	S (mm)	Dam Section	Points	R	S (mm)
1#	PL1-1	0.987	0.217	13#	PL13-2	0.997	0.912
	PL1-2	0.974	0.360		PL13-3	0.999	0.573
	IP1-1	0.953	0.360		PL13-4	0.996	0.787
5#	PL5-1	0.997	0.599		PL13-5	0.990	0.765
	PL5-2	0.998	0.423		IP13-2	0.984	0.364
	PL5-3	0.997	0.395	16#	PL16-1	0.993	1.014
	PL5-4	0.980	0.623		PL16-2	0.996	0.871
	IP5-1	0.910	0.091		PL16-3	0.997	0.707
9#	PL9-1	0.995	1.268		PL16-4	0.994	0.837
	PL9-2	0.997	0.931		PL16-5	0.990	0.703
	PL9-3	0.998	0.507		IP16-1	0.980	0.477
	PL9-4	0.999	0.290	19#	PL19-1	0.992	0.629
	PL9-5	0.986	0.275		PL19-2	0.996	0.511
	IP9-1	0.935	0.130		PL19-3	0.994	0.583
11#	PL11-1	0.998	0.964		PL19-4	0.988	0.691
	PL11-2	0.998	0.840		PL19-5	0.983	0.509
	PL11-3	0.998	0.674		IP19-1	0.988	0.183
	PL11-4	0.997	0.667	23#	PL23-1	0.955	0.339
	PL11-5	0.985	0.966		PL23-2	0.954	0.276
	IP11-1	0.969	0.573		PL23-3	0.954	0.261
13#	PL13-1	0.998	0.764		IP23-1	0.957	0.148

**Table 8:** Deformation hybrid model coefficients of typical measuring points.

Points	$\;a_{0}\;$	a	$\;b_{11}\;$	$\;b_{21}\;$	$\;b_{12}\;$	$\;b_{22}\;$	$\;c_{1}\;$	$\;c_{2}\;$
PL1-1	4.392	4.196	0.904	−0.065	−0.250	0.033	0.046	0.867
PL1-2	4.463	17.707	−0.330	−0.494	0.267	0.076	0.018	1.198
IP1-1	−0.278	−18.696	−0.249	−0.244	0.000	0.000	0.057	0.641
PL5-1	0.863	0.835	3.137	−1.786	−0.288	0.000	0.247	−1.770
PL5-2	1.135	0.880	−1.275	−1.746	0.236	−0.165	0.167	0.123
PL5-3	0.505	1.081	1.460	−0.189	−0.323	−0.045	0.152	0.506
PL5-4	0.780	2.074	1.295	−0.846	−0.466	0.061	0.179	0.792
IP5-1	0.099	−1.148	0.021	0.015	0.000	−0.022	0.030	0.000
PL9-1	−8.326	0.771	3.731	−3.361	0.000	−0.172	0.124	−4.058
PL9-2	−5.862	0.726	−1.491	−2.966	0.374	−0.096	0.052	−2.212
PL9-3	−2.818	0.755	1.653	0.690	0.000	−0.165	0.020	−1.022
PL9-4	−0.753	0.859	1.197	−0.177	−0.169	−0.085	0.055	−0.098
PL9-5	0.979	1.197	0.392	−0.246	−0.141	0.000	−0.025	0.383
IP9-1	0.409	14.357	−0.045	0.185	0.000	−0.020	0.000	0.230
PL11-1	−8.068	0.712	4.164	−2.939	−0.313	−0.137	0.231	−3.201
PL11-2	−5.984	0.692	−2.058	−3.465	0.614	−0.188	0.255	−1.764
PL11-3	−3.720	0.737	−2.389	−1.823	0.189	−0.264	0.167	0.233
PL11-4	0.074	0.778	−1.948	−0.747	0.000	−0.221	0.124	1.347
PL11-5	−1.694	0.984	1.586	−0.735	−0.412	0.000	0.244	0.897
IP11-1	−1.611	1.777	0.585	−0.336	−0.233	0.000	0.151	0.751
PL13-1	−11.022	0.772	3.021	3.952	0.000	0.000	0.000	−3.316
PL13-2	−4.555	0.659	−1.619	−3.565	0.537	−0.204	0.000	−2.540
PL13-3	−3.820	0.727	3.034	0.528	−0.271	−0.271	0.068	−1.117
PL13-4	−2.553	0.814	2.800	−0.781	−0.600	−0.076	0.095	−0.282
PL13-5	−0.619	0.905	1.736	−0.895	−0.597	0.000	0.070	0.564
IP13-2	−0.325	1.310	0.721	−0.373	−0.342	0.000	0.066	0.376
PL16-1	−2.132	0.811	3.428	2.279	0.505	0.132	0.111	−0.386
PL16-2	−2.338	0.719	−1.945	−2.472	0.634	−0.299	0.364	−1.083
PL16-3	−1.833	0.817	2.780	−0.268	−0.382	−0.237	0.254	−0.309
PL16-4	−1.494	0.960	2.676	−1.275	−0.681	0.000	0.295	0.201
PL16-5	0.442	1.078	1.614	−1.089	−0.539	0.100	0.136	1.103
IP16-1	−0.790	2.520	0.980	−0.784	−0.371	0.092	0.140	0.628
PL19-1	3.249	0.931	2.142	−1.333	−0.133	0.000	0.330	−1.575
PL19-2	0.632	0.873	−0.898	−1.296	0.183	−0.228	0.321	−0.102
PL19-3	−0.160	1.052	1.460	−0.413	−0.475	−0.098	0.363	0.429
PL19-4	−0.071	1.434	1.373	−1.010	−0.537	0.000	0.232	0.946
PL19-5	−0.496	2.272	0.829	−0.607	−0.369	0.000	0.229	0.549
IP19-1	−1.162	8.240	0.266	−0.152	−0.127	0.030	0.159	0.052
PL23-1	5.279	1.038	−0.937	−0.751	−0.200	−0.269	−0.115	0.585
PL23-2	−2.116	−9.697	0.180	0.235	−0.104	0.060	−0.082	0.865
PL23-3	1.108	−5.721	0.111	0.000	−0.125	0.041	−0.030	0.707
IP23-1	0.272	−7.884	0.266	−0.225	−0.122	0.000	0.008	0.294

**Table 9:** Spatial location of measurement points in spatiotemporal hybrid model.

Dam Section	Points	X (m)	Z (m)
1#	PL1-1	792.00	1885.00
	PL1-2	792.83	1830.00
	IP1-1	791.89	1778.00
5#	PL5-1	867.72	1885.00
	PL5-2	873.42	1830.00
	PL5-3	874.09	1778.00
	PL5-4	875.63	1730.00
	IP5-1	873.86	1601.00
9#	PL9-1	931.02	1885.00
	PL9-2	930.05	1830.00
	PL9-3	929.17	1778.00
	PL9-4	936.48	1730.00
	PL9-5	935.37	1664.25
	IP9-1	934.83	1601.00
11#	PL11-1	991.16	1885.00
	PL11-2	990.45	1830.00
	PL11-3	990.02	1778.00
	PL11-4	989.09	1730.00
	PL11-5	989.53	1664.25
	IP11-1	990.06	1601.00
13#	PL13-1	1030.26	1885.00
	PL13-2	1022.98	1830.00
	PL13-3	1022.92	1778.00
	PL13-4	1022.66	1730.00
	PL13-5	1022.49	1664.25
	IP13-2	1022.49	1601.00
16#	PL16-1	1104.84	1885.00
	PL16-2	1105.03	1830.00
	PL16-3	1105.42	1778.00
	PL16-4	1106.21	1730.00
	PL16-5	1105.78	1664.25
	IP16-1	1105.46	1601.00
19#	PL19-1	1162.66	1885.00
	PL19-2	1163.91	1830.00
	PL19-3	1162.97	1778.00
	PL19-4	1162.41	1730.00
	PL19-5	1161.26	1664.25
	IP19-1	1160.83	1601.00
23#	PL23-1	1224.58	1885.00
	PL23-2	1224.81	1778.00
	PL23-3	1224.87	1730.00
	IP23-1	1224.18	1601.00

**Table 10:** Correlation coefficient and residual standard deviation of spatiotemporal hybrid model.

Points	R	S (mm)
PL1-1~IP23-1	0.981	2.305

**Table 11:** Coefficients of spatiotemporal hybrid model of typical measuring points ($a_{0}=-1.04$)

$A_{ln}$ (l,n=0,1,2,3)	A00	A10	A20	A30	$\;B_{jkln}\;$ (j,k=0,1,2; l,n=0,1,2,3)	B2002	B2012	B2022	B2032
	−30.28	78.24	−65.37	18.70		3.40	−188.27	175.15	0.00
	A01	A11	A21	A31		B2003	B2013	B2023	B2033
	182.18	−453.85	354.67	−86.87		−3.46	143.39	−160.98	24.75
	A02	A12	A22	A32		B0200	B0210	B0220	B0230
	−348.74	873.52	−685.64	166.01		0.69	0.00	−2.86	2.14
	A03	A13	A23	A33		B0201	B0211	B0221	B0231
	219.08	−550.23	440.76	−110.75		−4.47	0.00	−64.79	64.27
$\;B_{jkln}\;$ (j,k=0,1,2; l,n=0,1,2,3)	B1000	B1010	B1020	B1030	$\;C_{jkln}\;$ (j,k=0,1,10; l,n=0,1,2,3)	B0202	B0212	B0222	B0232
	0.62	0.00	−2.80	3.03		3.84	80.97	0.00	−75.78
	B1001	B1011	B1021	B1031		B0203	B0213	B0223	B0133
	−4.91	0.00	−132.14	140.08		0.00	−86.86	82.62	0.00
	B1002	B1012	B1022	B1032		C1000	C1010	C1020	C1030
	4.10	137.32	150.19	−286.18		0.69	−1.09	0.00	0.32
	B1003	B1013	B1023	B1033		C1001	C1011	C1021	C1031
	0.00	−144.29	0.00	135.92		−3.50	−20.63	42.23	−18.09
	B0100	B0110	B0120	B0130		C1002	C1012	C1022	C1032
	−3.51	−6.75	31.99	−21.86		4.71	82.35	−146.21	60.40
	B0101	B0111	B0121	B0131		C1003	C1013	C1023	C1033
	26.84	0.00	245.33	−230.63		−2.02	−62.25	108.21	−45.47
	B0102	B0112	B0122	B0132		C0100	C0110	C0120	C0130
	−36.36	−271.01	−190.06	417.36		−11.10	42.54	−46.72	18.96
	B0103	B0113	B0123	B0133		C0101	C0111	C0121	C0131
	13.24	299.10	−142.85	−130.61		56.73	86.55	−38.55	−132.84
	B2000	B2010	B2020	B2030		C0102	C0112	C0122	C0132
	0.000	−10.13	24.36	−14.40		−64.47	−755.24	856.22	0.00
	B2001	B2011	B2021	B2031		C0103	C0113	C0123	C0133
	0.00	61.48	−55.41	0.00		22.08	635.42	−792.72	127.82

## Data Availability

The data used to support the findings of this study are available from the corresponding author upon request.
